# Developing and testing a nurse-led intervention to support bereavement in relatives in the intensive care (BRIC study): a protocol of a pre-post intervention study

**DOI:** 10.1186/s12904-020-00636-8

**Published:** 2020-08-18

**Authors:** Margo M. C. van Mol, Sebastian Wagener, Jos M. Latour, Paul A. Boelen, Peter E. Spronk, Corstiaan A. den Uil, Judith A. C. Rietjens

**Affiliations:** 1 Department of Intensive Care Adults, Erasmus MC University Medical Center, P.O. Box 2040, Room Ne409, 3000 CA Rotterdam, the Netherlands; 2 School of Nursing and Midwifery, Faculty of Health: Medicine, Dentistry and Human Sciences, University of Plymouth, Plymouth, UK; 3 Clinical Psychology Faculty Social Sciences, Arq Psychotrauma Expert Groep, University Utrecht, Utrecht, Netherlands; 4 Department of Intensive Care Medicine, ExpIRA - Expertise Center for Intensive Care Rehabilitation Apeldoorn, Gelre Hospitals Apeldoorn, Apeldoorn, The Netherlands; 5 Department of Cardiology, Erasmus MC University Medical Center, Rotterdam, the Netherlands; 6 Department of Public Health, Erasmus MC University Medical Center, Rotterdam, the Netherlands

**Keywords:** Bereaved relatives, Complicated grief, Intensive care unit, Nurse-led, Palliative care, Study protocol

## Abstract

**Background:**

When a patient is approaching death in the intensive care unit (ICU), patients’ relatives must make a rapid transition from focusing on their beloved one’s recovery to preparation for their unavoidable death. Bereaved relatives may develop complicated grief as a consequence of this burdensome situation; however, little is known about appropriate options in quality care supporting bereaved relatives and the prevalence and predictors of complicated grief in bereaved relatives of deceased ICU patients in the Netherlands. The aim of this study is to develop and implement a multicomponent bereavement support intervention for relatives of deceased ICU patients and to evaluate the effectiveness of this intervention on complicated grief, anxiety, depression and posttraumatic stress in bereaved relatives.

**Methods:**

The study will use a cross-sectional pre-post design in a 38-bed ICU in a university hospital in the Netherlands. Cohort 1 includes all reported first and second contact persons of patients who died in the ICU in 2018, which will serve as a pre-intervention baseline measurement. Based on existing policies, facilities and evidence-based practices, a nurse-led intervention will be developed and implemented during the study period. This intervention is expected to use 1) communication strategies, 2) materials to make a keepsake, and 3) a nurse-led follow-up service. Cohort 2, including all bereaved relatives in the ICU from October 2019 until March 2020, will serve as a post-intervention follow-up measurement. Both cohorts will be performed in study samples of 200 relatives per group, all participants will be invited to complete questionnaires measuring complicated grief, anxiety, depression and posttraumatic stress. Differences between the baseline and follow-up measurements will be calculated and adjusted using regression analyses. Exploratory subgroup analyses (e.g., gender, ethnicity, risk profiles, relationship with patient, length of stay) and exploratory dose response analyses will be conducted.

**Discussion:**

The newly developed intervention has the potential to improve the bereavement process of the relatives of deceased ICU patients. Therefore, symptoms of grief and mental health problems such as depression, anxiety and posttraumatic stress, might decrease.

**Trial registration:**

Netherlands Trial Register Registered on 27/07/2019 as NL 7875, www.trialregister.nl

## Background

### Bereavement in the intensive care unit

The integration of bereavement care into the support services offered to relatives of intensive care unit (ICU) patients has been described and endorsed by national and international ICU societies [1–4]. When a patient is approaching death in the ICU, relatives need to make a rapid transition from focusing on the recovery of their beloved one to preparing for their unavoidable death. The actual risk of death depends on the underlying disease and may surpass 50% in high-risk ICU patients [5]. Withholding and/or withdrawing life-sustaining measures in those patients has become common practice preceding death among patients in ICUs worldwide, with frequencies varying within and between countries from 1.7 to 85% [6]. Death might occur within minutes to days after the initiation of withdrawing life-sustaining therapy [7]. Furthermore, the time of death is sometimes postponed, for example, in the situation of an organ donation procedure or to provide the relatives some extra time to say goodbye to their loved one. Therefore, a patient’s death in an ICU can have a strikingly guided character and is difficult to compare with the dying situations in other healthcare settings or at home [8]. Palliative care in this phase aims to improve the quality of dying and death with personalized attention to physical, social, psychological and spiritual dimensions of care and well-being [2, 9], using variable methods and care plans [10]. Supporting the bereavement process of relatives during the ICU admission of their dying loved one has been incorporated into the daily care offerings of professionals worldwide [9, 11]. However, a gap exists in adequate ICU-based studies evaluating family-centered experiences and long-term health outcomes of bereavement care other than ‘satisfaction’ after the death of an ICU patient [9].

### Long-term grief in bereaved relatives

Grieving, with intense feelings and behavior of mourning, is a normal emotional reaction to the loss of a meaningful loved one and refers to the transition from the experience of loss to the adaptation to it [12]. Grieving is not restricted to specific thoughts, feelings, and behaviors, nor is it restricted to a limited time period. Grief after a sudden and unexpected death of a beloved person in the ICU is going to be hard and will probably last longer than 6 months. A wide variety of phenomena impede the establishment of complicated grief, which is described as serious and persistent grief with adjustment problems in the long term [13]. The Diagnostic and Statistical Manual of Mental Disorders (DSM) 5th edition defines ‘persistent complex bereavement disorder’ while the International Classification of Diseases (ICD) -11 includes ‘prolonged grief disorder’ as practically the same diagnostic entity, differing merely semantically [14]. This disorder is, among other signs, characterized by intense symptoms of grief lasting for more than 6 months post-loss, separation distress, intrusive thoughts, and feelings of emptiness or meaninglessness [13]. A recent meta-analysis revealed a prevalence of approximately 10% for grieving disorders among bereaved adults in a general population [15] which could lead to negative health outcomes, increased medical service utilization, and economic cost due to absenteeism from work [16].

Relatives of deceased ICU patients may develop complicated grief as a consequence of the unpredictable and burdensome situation of losing their loved one. Therefore, complicated grief has been included in the Post Intensive Care Syndrome-Family (PICS-F) framework [17, 18]. Demographic variables such as gender, relationship status, and cultural background, might be associated with complicated grief [19, 20]. In addition, factors related to quality of dying and death, communication of staff, and bereavement care might impact the process of grieving for ICU relatives [21, 22]. However, little is known about the determinants and actual prevalence of complicated grief in bereaved relatives of deceased ICU patients. In two small single-center studies, the prevalence of complicated grief measured by the Inventory of Complicated Grief (ICG) ranged from 5% (two out of 41) [23] to 46% (six out of 13) [24]. A French multi-center study among the relatives of 475 deceased ICU patients reported an incidence of complicated grief assessed by the ICG in half of the respondents at 6 months (52%), which remained unchanged at 12 months [19]. The same study presented a decline in posttraumatic stress symptoms from 6 to 12 months, 44 and 36% respectively, as measured by the Impact of Event Scale-Revised (IES-R). Despite the robust study design, the generalizability of these results to other countries remains unclear due to cultural differences in end-of-life perspectives and subsequent bereavement care. To our knowledge only one study has measured the experiences of bereaved relatives in a Dutch ICU [8]. Among 51 respondents the most reported complaints were sleeping problems, while 86% returned to work and normal activities within 4–16 months. Although this study did not measure complicated grief nor symptoms of stress, it reflects the normal grieving reactions being hard and lasting for a longer period for bereaved relatives in the ICU [8]. Patients could die rapidly, resulting in a dignified death with a low burden of suffering and few signs of discomfort [6]. A Dutch study found that the quality of the dying and death process was perceived as being high by both care-givers and relatives [25]. Therefore, it is important to explore grief and the experiences with the quality of bereavement care in relatives of deceased ICU patients in the Netherlands compared to the existing international findings.

### Supporting relatives of deceased ICU patients

To support relatives during ICU admission, multidisciplinary ICU teams have previously developed supporting interventions in the bereavement process [21, 26, 27]. Effective communication between relatives and ICU professionals, professionals’ empathic attitudes, and personalized interactions are highly valued aspects of care in the relatives’ perspective, particularly during the dying and death process of the patient [26, 28]. These aspects may improve preparedness for the expected death and should be tailored to the relatives’ specific needs [29]. In addition, bereaved relatives may need follow-up services to discuss the patient’s suffering or distress, to find answers to any remaining questions regarding the death of their beloved one, to discuss their own feelings of loneliness and to explore their expectations for the future [30]. Relatives also reported a preference for more formal support for their emotional situation and psychological symptoms in the early bereavement period (< 6 months) [31]. According to a European study among ICU nurses from 18 countries, bereavement follow-up services vary between countries and ICUs, such as ‘viewing the dead patient in the unit’ (91%) and ‘a phone call at a certain timepoint’ (31.0%) [32]. This international group of experts suggested in their study to further explore the needs of the relatives, to test the efficacy of interventions in bereavement care and to develop guidelines for ICUs aiming to adequately deliver support to relatives during this difficult situation.

### Aim

The aims of the BRIC (Bereavement in Relatives in the Intensive Care) study for bereaved relatives of deceased ICU patients are: 1) to develop and implement a multicomponent nurse-led intervention, 2) to explore the experiences with bereavement care such as aspects of communication, quality of ding and death, and quality of support to relatives, 3) to determine the effectiveness of this intervention on complicated grief, anxiety, depression and posttraumatic stress and 4) to identify determinants and risk factors of complicated grief.

### Hypotheses

We hypothesize the following:

1. A newly developed multicomponent nurse-led intervention, including communication strategies, materials to make a keepsake, and a nurse-led follow-up service, improves the quality of experiences with bereavement care in the ICU from the perspectives of the bereaved relatives.
2. Symptoms of complicated grief depression, anxiety and posttraumatic stress, decrease after implementation of the multicomponent nurse-led intervention.

## Methods

The BRIC study is a cross-sectional pre-post intervention study. Two different consecutive groups of bereaved relatives, cohort 1 and 2, will be approached to participate in a single-site study to compare complicated grief, anxiety, depression, posttraumatic stress and experiences with bereavement care. Cohort 1 will receive the standard of care, including ICU nurses’ presence and support for relatives, explanation of the process of dying and death, and empathic communication skills. Cohort 2 will receive additional bereavement support through a newly developed multicomponent nurse-led intervention.

### Setting

The study setting is a university hospital in the Netherlands with a 48-bed mixed ICU divided into four units.

### Study population

The study population consists of bereaved relatives after the death of an adult (age ≥ 18) ICU patient who fulfil all inclusion criteria and none of the exclusion criteria. Hospital records are used to identify the patients’ relatives, i.e., the first and second contact persons. Respondents will be selected using four criteria: 1) their loved one had died in the predefined period; 2) they were present during the ICU stay preceding death; 3) they have sufficient knowledge of the Dutch language (to read and understand information on the study and the questionnaires); and 4) they are considered legally responsible. Relatives with unknown contact details will be excluded.

### Methodology

This study protocol follows the Standard Protocol Items: Recommendations for Interventional Trials (SPIRIT) checklist [33]. The recommended schematic diagram detailing the schedule of enrolment, interventions and assessments is provided in Table 1. The study is non-blinded and non-randomized due to practical issues in the multicomponent intervention elements, which were applied intuitively and in a tailored fit to the relatives by ICU nurses.

**Table 1:**
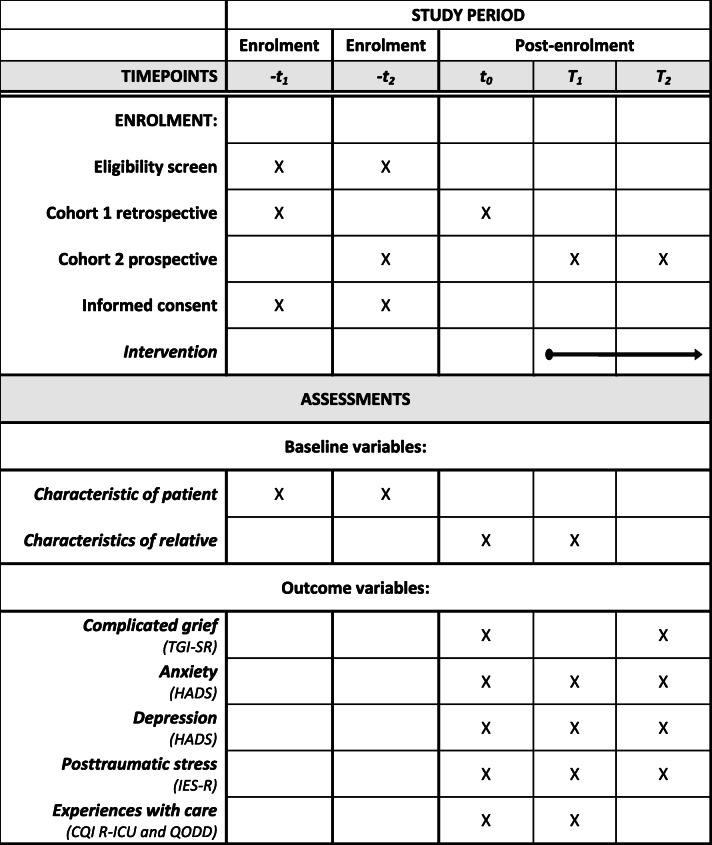
Schedule of enrolment, intervention, and assessments according SPIRIT figure

BRIC Bereavement in Relatives in the Intensive Care; −t_1_ Approach and enrolment of cohort 1; −t_1_ Approach and enrolment of cohort 2; t_0_ Baseline measurement; t_1_ 6 to 8 weeks post-loss; t_2_ 6 months post-loss; TGI-SR Traumatic Grief Inventory-Self Report Version’; HADS Hospital Anxiety and Depression Scale; IES-R ‘Impact of Events Scale-Revised’; CQI R-ICU ‘Consumer Quality Index Relatives in the ICU’; QODD ‘The Quality of Dying and Death questionnaire’

#### Procedure

Consecutive ICU patients dying in the ICU between January 2018 and March 2020 will be identified from the ICU database. Descriptive data such as age, gender, length of stay, cause of illness and date of death will be extracted. Eligible relatives will be approached by telephone, where they are informed about the study and invited to participate in a survey exploring their current health situation and experiences with the quality of bereavement care. If interested, contact details such as email or postal address will be gathered to send additional written information and an informed consent form. After receiving the bereaved relative’s signed consent form, questionnaires will be send according to the participants’ preference of a digital version of the questionnaires or paper version with a stamp-free envelope.

#### Multicomponent nurse-led bereavement support intervention

Based on existing (inter) national policies, facilities and evidence-based practices [34], we aim to develop a nurse-led support intervention with subsequent implementation in practice during the study period. This intervention is expected to include 1) communication strategies, such as an information leaflet on loss and grief [21], a condolence greeting card [35], and a checklist with relevant topics to discuss in the process of dying and death [26]; 2) materials to make a keepsake [32], such as a lock of hair and a fingerprint; and 3) a nurse-led follow-up service, such as a memorial meeting and telephone follow-up calls 4 months post-loss [36]. The intervention will be developed in a multidisciplinary collaboration, including intensivists, spiritual service and social workers. The nurses will be invited to extend the standard of care with elements of the intervention applied to the needs and values of the relatives, thus providing personalized bereavement support. This multicomponent nurse-led intervention will be developed in co-creation with bereaved relatives to maximize care according to their ideals and perspectives, and not solely built on professional beliefs. Therefore, the Dutch foundation ‘Family and patient Centered Intensive Care’ (FCIC) will be involved in the development process.

To strengthen the development and implementation of the intervention fidelity measures will be used which will help to understand whether planned intervention was effective [37]. Several methods can be used assessing the quality and include both acceptability of measures in relation to the needs of the stakeholders (bereaved relatives) and practicality of the measures in relation to applicability for the users (ICU professionals). The implementation evaluation of this complex intervention will be performed by applying the RE-AIM model; Reach, Efficacy, Adoption, Implementation and Maintenance. The RE-AIM model is an instrument that measures the total impact of an intervention and provides insight into causes for (in)efficacy. RE-AIM is widely used in public health research, and will support the applicability and dissemination of the study results [38]. Table 2 provides an overview of the RE-AIM model applied in the current study.

**Table 2:** Overview of the RE-AIM model applied in the BRIC study

RE-AIM	Characteristics	Level	Data collection
Reach	Baseline characteristics	Individual	Demographic data cohort 1 and 2
	Inclusion rate	Individual	Medical files
Efficacy	Comparing the study outcomes (before, after; corrected for covariates)	Individual	Measurements in cohort 1 and 2
Adoption	Proportion of ICU professionals using intervention elements	Organizational	Self-composed questionnaire among ICU professionals
Implementation	Number of intervention elements received by relatives	Individual	Self-composed items added to questionnaire measured in cohort 2
	Experiences with implementation	Organizational	Self-composed questionnaire among ICU professionals
Maintenance	Long-term adoption of the intervention	Organizational	Semi-structured interview with ICU manager

#### Study cohorts and time points

**Fig. 1:**
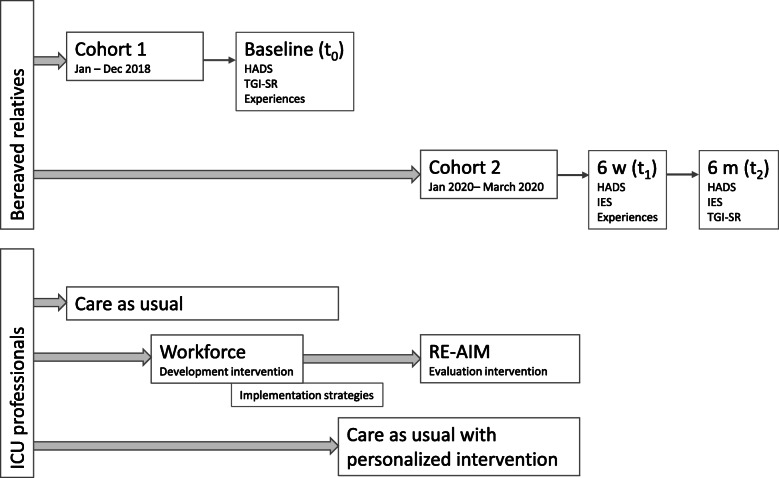
BRIC study design. BRIC Bereavement in Relatives in the Intensive Care; t_0_ baseline measurement; t_1_ 6 to 8 weeks post-loss; t_2_ 6 months post-loss; TGI-SR Traumatic Grief Inventory-Self Report Version’; HADS Hospital Anxiety and Depression Scale; IES-R ‘Impact of Events Scale-Revised’; CQI R-ICU ‘Consumer Quality Index Relatives in the ICU’; QODD ‘The Quality of Dying and Death questionnaire’; RE-AIM model; Reach, Efficacy, Adoptation, Implementation and Maintenance

Both cohorts will be performed in study samples of 200 relatives per group, all participants will be invited to complete questionnaires measuring complicated grief, anxiety, depression and posttraumatic stress. Cohort 1 includes all reported first and second contact persons of patients who died in the ICU in 2018, which will serve as a pre-intervention baseline measurement. Eligible relatives were approached from March to May 2019 (−t_1_) and participants received the questionnaires directly after they signed the consent form (t_0_). The intervention was conducted from June 2019 onwards. Cohort 2, including all bereaved relatives in the ICU from October 2019 until March 2020, will serve as a post-intervention follow-up measurement. Eligible relatives will be approached 12 weeks after the death of their loved one (−t_2_) and participants will receive the questionnaires directly (t_1_) and at 6 months (t_2_) after they signed the consent form. The study design and timeline is presented in Fig. 1. For each deceased patient, up to 3 relatives can be included in the study. This recruitment process has been shown feasible in a previous study [39].

#### Primary outcomes

Complicated grief will be measured with the Dutch version of the ‘Traumatic Grief Inventory-Self Report Version’ (TGI-SR), 18 items [40]. Respondents are asked to rate the extent to which they experienced the 18 symptoms listed during the preceding month on a 5-point scale: 1 = ‘never,’ 2 = ‘rarely,’ 3 = ‘sometimes,’ 4 = ‘frequently,’ and 5 = ‘always’. The TGI-SR demonstrated strong internal consistency, Cronbach’s alpha = .95. A total TGI-SR score, providing an index of the severity of potentially problematic grief, can be obtained by summing the 18 items. A total symptom severity score (range 17–85) can be obtained by summing the scores for items 1–11 and 13–18. Elevated scores (tentatively, a cut-off score of ≥61 meets the criteria for a provisional diagnosed grief disorder) correlate significantly with elevated scores on indices of psychopathology and lower quality of life, attesting to the concurrent validity [40]. Therefore, in our study, the risk of complicated grief will be categorized as ‘low risk’ (17 to 60) or ‘high risk’ (≥ 61).

#### Secondary outcomes

- Anxiety and depression; measured with the Dutch version of the ‘Hospital Anxiety and Depression Scale’ (HADS), that includes to 7 items on the subscales tapping ‘Anxiety’ and ‘Depression’ respectively [41, 42]. These subscales are reliable and valid measures of mental health status with items concerning symptoms of psychological well-being. Scores range from 0 to 21, categorized as ‘normal’ (0 to 7); ‘mild’ (8 to 10); and ‘moderate to severe’ (11–21).
- Posttraumatic stress; measured with the 21-item Dutch version of the ‘Impact of Events Scale-Revised’ (IES-R) [43]. This measuring instrument is used worldwide to self-report the frequency of intrusive and avoidant phenomena after a variety of traumatic experiences. The reliability of the Dutch version of the IES is adequate across the various stressors [44]. Scores range from 0 to 88, categorized as ‘low risk’ (0 to 11); ‘moderate risk’ (12 to 32); and ‘high risk’ (≥ 33).
- Experiences with care; measured with items derived from the ‘Consumer Quality Index Relatives in the ICU’ (CQI R-ICU) [45] and ‘The Quality of Dying and Death questionnaire’ (QODD) [46, 47], includes 30 items in total. Both instruments have been developed and validated in Dutch, and report high internal consistency reliability and construct validity. The subscales measure aspects of communication, quality of dying and death, and quality of support to relatives.
- Questions to evaluate which intervention elements were actually received and how they were appreciated will be added to the questionnaires for cohort 2.
- The applicability and opinions of ICU professionals will be measured with a self-composed questionnaire including items to evaluate the fit of the intervention to daily practice and to assess the implementation process. Also, a semi-structured interview with one ICU manager will be performed for deepening the evaluation and describe learned lessons before further dissemination. These measures administered to ICU staff will be performed between October 2019 and March 2020, when the development and implementation of the nurse-led multicomponent intervention to support bereavement in relatives in the IC has been finished*.*

#### Data handling

Data will be collected using Limesurvey (Version 2.06lts Build 160,524) and exported to a secure SPSS database (© IBM SPSS Statistics for Windows, Version 25.0. Armonk, NY: IBM Corp) for management and analysis. All principal investigators will have access to the final study dataset, of which one delegate has control over study codes with links from personal data of the patients and their relatives. To avoid potential bias, the researchers will be blinded from any results that can relate data back to the individual respondents. Personal data will be anonymised.

#### Statistical analysis

Power and feasibility: Baseline (retrospective; t_1_) and follow-up measurements (6 weeks; t_2_ and 6 months; t_3_) will be performed in study samples of 200 relatives per group. These numbers are feasible, starting from a mean of 325 ICU deaths each year in the study setting, given a (2x) 12-month inclusion period, an expected 1.5 loved one per death, and an expected inclusion rate of 50% at 6 weeks and a retention rate of 80% at 6 months. These numbers ensure an effect size of 0.5 with a GPower t-test for the ´difference between two independent means´ (1-β = 95%, *p* < .05, two-tailed, d = .5) [48].

Analyses: Descriptive statistics (e.g., means, medians, or proportions as appropriate) and Student’s T-test between pre-post groups on demographic variables (e.g., gender, age, educational level) and outcome measures (e.g., complicated grief, anxiety, depression, posttraumatic stress, experiences with care) will be used to present noticeable differences between the baseline and intervention groups. Missing data will be handled using the multiple imputation. The scores will be analysed based on original data, and when available, according to established cut-offs. All test will be bilateral and significance will be defined as *p* < .05.

To test the hypotheses repeated measure analyses of variance (ANOVA) will be conducted with time as the within-subject factor (pre- versus post-intervention) and group (cohort 1 and 2) as the between-subject factor. Cohen’s d will be calculated to present effect sizes if applicable. Hierarchical regression analyses will be performed to explore determinants and identify subgroups (such as gender, ethnicity, risk profiles, relationship with patient) of bereaved relatives in the ICU who are at particular risk of developing mental health problems (i.e., those with scores above established cut-offs). Covariates, such as reason of admission, severity of illness, cause of death, bereavement care, time to say goodbye and social support, will be included in the regression model. This model will be adapted for cluster effects to correct for multiple relatives per deceased patient.

#### Study status

The study is currently ongoing with recruiting relatives, which started with cohort 1 on March 1st 2019. Subsequently, the intervention has been developed as scheduled (Fig. 1) and implemented in daily practice accordingly. Recruiting of respondents for cohort 2 has been postponed because of the COVID-19 pandemic and will start immediately after management consent.

## Discussion

Providing bereavement care to relatives in the ICU is an important part of palliative care. Alongside a temporary disruption in their personal life, grief might have negative social and economic consequences as well, such as reduced time at work and decreased income. In today’s society and culture, talking about death is not always taken for granted. The added value of this research project is to improve psychosocial care for relatives during and after the death of their loved one. The newly developed multicomponent intervention may improve the bereavement process of the relatives; therefore, symptoms of complicated grief and related mental health problems of PICS-F such as depression, anxiety and posttraumatic stress, might decrease. While most bereaved relatives do not require bereavement support from a specialist (such as psychologist, psychiatrist), a considerable minority will benefit from non-specialized support (e.g., mutual help-groups) [49]. Identifying relatives at risk for mental health problems, will help to recognize the need for specialized support in an early stage. Another strength of the current study is the retrospective baseline measurement combined with a longitudinal prospective approach, thus collecting data in several timepoints to assess changes in mental health over time. These findings can inform ICU professionals on strategies to build an evidence-based guideline in bereavement services [32].

### Limitations and related risk strategies

This single-site study, based on the self-reported questionnaire answers of the respondents, may provoke bias in the results and limit generalizability. However, comparing the results with previous findings and international literature will minimize inappropriate conclusions. A stepped wedge or cluster randomized study could provide more general results, which is not the case in this study due to grant requirement of the institution. Another limitation may be difficulties with the implementation of multicomponent intervention. The adherence of the professionals may be influenced by workload pressure in clinical practice, insufficient knowledge, focus on high-tech and medical priorities, and their own vulnerability in delicate situations [50]. Moreover, ICU nurses may experience barriers in knowledge and competences in communication during the end-of-life situation [51]. Oncology nurses have built a broad expertise in the signs of complicated grief [52], which can provide a starting point to support the educational needs of ICU nurses [53].

Six strategies have been developed to stimulate the usage of the multicomponent intervention and limit the risk of non-adherence [31, 54–56]:

- Educational sessions for all ICU nurses presenting the new tools and discussing communication strategies;
- Information strategies such as an informational pamphlet and reminders in a weekly newsletter;
- Champions in each ICU team, empowered by a two-day training in loss and bereavement care;
- Regular interactions between the investigators, the local champions and the team members to discuss difficulties;
- Including the nurse managers in advocating the bereavement tools if doomed necessary during daily start-up;
- Close collaboration with the department of Public Health and Erasmus MC University Medical Center, with extended expertise in this domain of palliative care and used practices among nurses and other allied healthcare providers.

### Ethical considerations

This particular area of research, and some items in the questionnaires specifically, may be a confronting issue for participants. Bereaved relatives are vulnerable, and even voluntary participation in the study might evoke negative flashbacks of the ICU admission and death of their loved one. This possibility is taken into account by allowing the participants to share their own experience when completing the questionnaires. They choose what to reveal, and they are not required to answer. Previous studies have shown that comparable respondents usually characterize their participation as helpful and not harmful [57, 58]. Furthermore, information about supporting services such as contact with the research team, social work, and an independent medical specialist, is included in the participant information form and at the beginning of the survey. Those who express a need for support will be brought into contact with a social worker, a psychologist or their general practitioner (GP). The GP will be informed in advance on participation of their patient in the current study by an information letter. The study protocol is approved by the Medical Ethics Committee of Erasmus MC (MEC-2018-1598). Protocol modifications will be communicated to the study sponsor by email and to the Medical Ethics Committee by protocol amendment.

### Data dissemination

Public access to the study protocol, study details, participant-level dataset, and statistical code can be obtained from the correspondence author. The results will be disseminated to healthcare professionals, health services authorities and the public via presentations at national and international meetings and published in peer-reviewed journals. A lay summary of the results will be written and shared with the Dutch foundation FCIC and made available to participants on request.

## Conclusion

An accurate assessment of the implementation process through the RE-AIM model, combined with a high degree of fidelity to the intervention, is critical to the reliability, validity, replicability, and scale-up of the results of an intervention research study. The findings and evaluation of this study will be used to design and conduct a future multicentre trial. Nurses from other ICUs and nurses of other subspecialties, such as cancer nursing, will be encouraged to implement the developed intervention and to study the effects gaining comparative data. Finally, guidelines will be developed for ICUs aiming to adequately deliver support to relatives during the process of dying and death.

## Data Availability

Anonymized data gathered and analysed during the current study are not publicly available due to legal and ethical restriction. These can be requested from the corresponding author as well as text and photo material of the developed intervention.

## Abbreviations

ANOVA: Analysis of Variance
DSM-5: Diagnostic and Statistical Manual of Mental Disorders
FCIC: Family and patient Centered Intensive Care
GP: General Practitioner
HADS: Hospital Anxiety and Depression Scale
ICD-11: International Classification of Diseases
ICG: Inventory of Complicated Grief
ICU: Intensive Care Unit
IES-R: Impact of Event Scale-Revisited
TGI-SR: Traumatic Grief Inventory-Self Report Version
PICS-F: Post Intensive Care Syndrome Family
PIF: Participant Information Form
RE-AIM: Reach, Efficacy, Adoptation, Implementation and Maintenance
SPIRIT: Standard Protocol Items: Recommendations for Interventional Trials

## References

[1] Myburgh J (2016). End-of-life care in the intensive care unit: report from the task force of world Federation of Societies of intensive and critical care medicine. J Crit Care.

[2] Davidson JE (2017). Guidelines for family-centered care in the neonatal, pediatric, and adult ICU. Crit Care Med.

[3] National Consensus Project for Quality Palliative Care (2004). Clinical practice guidelines for quality palliative care. Kansas Nurse.

[4] Noome M (2017). Effectiveness of supporting intensive care units on implementing the guideline ‘end-of-life care in the intensive care unit, nursing care’: a cluster randomized controlled trial. J Adv Nurs.

[5] NICE. Basic data ICUs in the Netherlands. [cited 2019; Available from: https://www.stichting-nice.nl/datainbeeld/public.

[6] Epker JL (2015). An observational study on a protocol for withdrawal of life-sustaining measures on two non-academic intensive care units in the Netherlands: few signs of distress, no suffering?. J Pain Symptom Manag.

[7] van Beinum A (2016). Exploration of withdrawal of life-sustaining therapy in Canadian intensive care units. J Intensive Care Med.

[8] van der Klink MA (2010). Survey into bereavement of family members of patients who died in the intensive care unit. Intensive Critical Care Nurs.

[9] Aslakson R, Spronk P. Tasking the tailor to cut the coat: how to optimize individualized ICU-based palliative care? Springer. 2016. 10.1007/s00134-015-4107-4 26556621

[10] Metaxa V (2019). Palliative care interventions in intensive care unit patients - a systematic review protocol. Syst Rev.

[11] Kentish-Barnes N (2016). CAESAR: a new tool to assess relatives’ experience of dying and death in the ICU. Intensive Care Med.

[12] Parkes CM (1988). Bereavement as a psychosocial transition: processes of adaptation to change. J Soc Issues.

[13] Prigerson HG, et al. Correction: Prolonged Grief Disorder: Psychometric Validation of Criteria Proposed for DSM-V and ICD-11. PLoS Med. 2013:10(12). 10.1371/journal.pmed.1000121 PMC2711304 19652695

[14] Hoffmann R (2018). Internet-based grief therapy for bereaved individuals after loss due to Haematological cancer: study protocol of a randomized controlled trial. BMC Psychiatry.

[15] Lundorff M (2017). Prevalence of prolonged grief disorder in adult bereavement: a systematic review and meta-analysis. J Affect Disord.

[16] Holland JM (2016). Prolonged grief symptoms related to loss of physical functioning: examining unique associations with medical service utilization. Disabil Rehabil.

[17] Harvey MA, Davidson JE (2016). Postintensive care syndrome: right care, right now … and later. Crit Care Med.

[18] Needham DM (2012). Improving long-term outcomes after discharge from intensive care unit: report from a stakeholders' conference. Crit Care Med.

[19] Kentish-Barnes N (2015). Complicated grief after death of a relative in the intensive care unit. Eur Respir J.

[20] Savelkoul C, et al. Culturally sensitive communication in end-of-life care: the care for Muslim patients as an example. Ned Tijdschr Geneeskd. 2017;161:D1410–0. 28745252

[21] Lautrette A (2007). A communication strategy and brochure for relatives of patients dying in the ICU. N Engl J Med.

[22] Kentish-Barnes N (2017). Effect of a condolence letter on grief symptoms among relatives of patients who died in the ICU: a randomized clinical trial. Intensive Care Med.

[23] Siegel MD (2008). Psychiatric illness in the next of kin of patients who die in the intensive care unit. Crit Care Med.

[24] Anderson WG (2008). Posttraumatic stress and complicated grief in family members of patients in the intensive care unit. J Gen Intern Med.

[25] Gerritsen RT (2013). Perception by family members and ICU staff of the quality of dying and death in the ICU: a prospective multicenter study in the Netherlands. Chest.

[26] Kentish-Barnes N, Chevret S, Azoulay E (2018). Guiding intensive care physicians’ communication and behavior towards bereaved relatives: study protocol for a cluster randomized controlled trial (COSMIC-EOL). Trials.

[27] Long AC, Curtis JR. Quality of dying in the ICU: understanding ways to make it better. Intensive Care Med. 2014;40(11):1793–3. 10.1007/s00134-014-3512-4 25288214

[28] Davidson JE (2009). Family-centered care: meeting the needs of patients’ families and helping families adapt to critical illness. Crit Care Nurse.

[29] Hebert RS (2006). Preparing caregivers for the death of a loved one: a theoretical framework and suggestions for future research. J Palliat Med.

[30] Milberg A (2008). Family members' perceived needs for bereavement follow-up. J Pain Symptom Manag.

[31] Downar J, Barua R, Sinuff T (2014). The desirability of an intensive care unit (ICU) clinician-led bereavement screening and support program for family members of ICU decedents (ICU Bereave). J Crit Care.

[32] Egerod I (2019). Elements of intensive care bereavement follow-up services: a European survey.

[33] Chan A-W (2013). SPIRIT 2013 explanation and elaboration: guidance for protocols of clinical trials. BMJ.

[34] Ågård AS (2019). Identifying improvement opportunities for patient-and family-centered care in the ICU: using qualitative methods to understand family perspectives. J Crit Care.

[35] Bedell SE, Cadenhead K, Graboys TB. The doctor's letter of condolence. Mass Medical Soc. 2001. 10.1056/NEJM200104123441510 11302139

[36] Kentish-Barnes N (2015). Research participation for bereaved family members: experience and insights from a qualitative study. Crit Care Med.

[37] Walton H (2020). Developing quality fidelity and engagement measures for complex health interventions. Br J Health Psychol.

[38] Glasgow RE, Vogt TM, Boles SM (1999). Evaluating the public health impact of health promotion interventions: the RE-AIM framework. Am J Public Health.

[39] Bruinsma S (2016). No negative impact of palliative sedation on relatives’ experience of the dying phase and their wellbeing after the Patient’s death: an observational study. PLoS One.

[40] Boelen PA, Smid GE (2017). The traumatic grief inventory self-report version (TGI-SR): introduction and preliminary psychometric evaluation. J Loss Trauma.

[41] Bjelland I (2002). The validity of the hospital anxiety and depression scale: an updated literature review. J Psychosom Res.

[42] Spinhoven P (1997). A validation study of the hospital anxiety and depression scale (HADS) in different groups of Dutch subjects. Psychol Med.

[43] Weiss DS. The impact of event scale: revised, in Cross-cultural assessment of psychological trauma and PTSD. Springer. 2007:219–38.

[44] van der Ploeg E (2004). Construct validation of the Dutch version of the impact of event scale. Psychol Assess.

[45] Rensen A (2017). Quality of care in the intensive care unit from the perspective of patient’s relatives: development and psychometric evaluation of the consumer quality index ‘R-ICU’. BMC Health Serv Res.

[46] Patrick DL, Engelberg RA, Curtis JR (2001). Evaluating the quality of dying and death. J Pain Symptom Manag.

[47] Gerritsen RT (2017). Comparing quality of dying and death perceived by family members and nurses for patients dying in US and Dutch ICUs. Chest.

[48] Faul F (2007). G* power 3: a flexible statistical power analysis program for the social, behavioral, and biomedical sciences. Behav Res Methods.

[49] Breen LJ, Aoun SM, O’Connor M (2015). The effect of caregiving on bereavement outcome: study protocol for a longitudinal, prospective study. BMC Palliat Care.

[50] van Mol MM (2017). Patient-and family-centred care in the intensive care unit: a challenge in the daily practice of healthcare professionals. J Clin Nurs.

[51] Crump SK, Schaffer MA, Schulte E (2010). Critical care nurses' perceptions of obstacles, supports, and knowledge needed in providing quality end-of-life care. Dimens Crit Care Nurs.

[52] Witkamp FE (2016). Effect of palliative care nurse champions on the quality of dying in the hospital according to bereaved relatives: a controlled before-and-after study. Palliat Med.

[53] Boyle, D.A., et al. Palliative care communication in the ICU: implications for an oncology-critical care nursing partnership. In Seminars in oncology nursing. 2017. Elsevier. 10.1016/j.soncn.2017.10.003 29107532

[54] Grol R, et al. Improving patient care: the implementation of change in health care: John Wiley & Sons; 2013.

[55] Efstathiou N (2019). The state of bereavement support in adult intensive care: a systematic review and narrative synthesis. J Crit Care.

[56] Bero LA (1998). Closing the gap between research and practice: an overview of systematic reviews of interventions to promote the implementation of research findings. BMJ.

[57] Williams BR (2008). Identifying and responding to ethical and methodological issues in after-death interviews with next-of-kin. Death Stud.

[58] Bentley B, O'Connor M (2015). Conducting research interviews with bereaved family carers: when do we ask?. J Palliat Med.

